# A117 VALIDITY EVIDENCE FOR ENDOSCOPIC RETROGRADE CHOLANGIOPANCREATOGRAPHY COMPETENCY ASSESSMENT TOOLS: A SYSTEMATIC REVIEW

**DOI:** 10.1093/jcag/gwac036.117

**Published:** 2023-03-07

**Authors:** R Khan, H Homsi, N Gimpaya, N Sabrie, R Gholami, R Bansal, M Scaffidi, D Lightfoot, P James, K Siau, N Forbes, S Wani, R Keswani, C Walsh, S Grover

**Affiliations:** 1 Western University, London; 2 St. Michael's Hospital; 3 University of Toronto, Toronto; 4 McMaster University, Hamilton; 5 Queen's University, Kingston; 6 University Health Network, Toronto, Canada; 7 University of Birmingham College of Medical and Dental Sciences, Birmingham, United Kingdom; 8 University of Calgary, Calgary, Canada; 9 University of Colorado Anschutz Medical Campus, Aurora; 10 Northwestern University, Chicago, United States; 11 The Hospital for Sick Children, Toronto, Canada

## Abstract

**Background:**

Assessment of competence in endoscopic retrograde cholangiopancreatography (ERCP) is essential to ensure trainees possess the skills needed for independent practice. Traditionally, ERCP training has used the apprenticeship model, whereby novices learn skills under the supervision of an expert. A growing focus on procedural quality, however, has supported the implementation of competency-based medical education models which require documentation of a trainee’s competence for independent practice. Observational assessment tools with strong evidence of validity are critical to this process. Validity evidence supporting ERCP observational assessment tools has not been systematically evaluated.

**Purpose:**

To conduct a systematic review of ERCP assessment tools and identify tools with strong evidence of validity using a unified validity evidence framework

**Method:**

We conducted a systematic search using electronic databases and hand-searching from inception until August 2021 for studies evaluating observational assessment tools of ERCP performance. We used a unified validity framework to characterize validity evidence from five sources: content, response process, internal structure, relations to other variables, and consequences. Each domain was assigned a score of 0-3 (maximum score 15). We assessed educational utility and methodological quality using the Accreditation Council for Graduate Medical Education framework and the Medical Education Research Quality Instrument, respectively.

**Result(s):**

From 2769 records, we included 17 studies evaluating 7 assessment tools. Five tools were studied for clinical ERCP, one on simulated ERCP, and one on simulated and clinical ERCP. Validity evidence scores ranged from 2-12. The Bethesda ERCP Skills Assessment Tool (BESAT), ERCP Direct Observation of Procedural Skills Tool (ERCP DOPS), and The Endoscopic Ultrasound (EUS) and ERCP Skills Assessment Tool (TEESAT) had the strongest validity evidence with scores of 10, 12, and 11, respectively. Regarding educational utility, most tools were easy to use and interpret, and required minimal additional resources. Overall methodological quality was strong, with scores ranging from 10-12.5 (maximum 13.5).

**Conclusion(s):**

The BESAT, ERCP DOPS, and TEESAT have strong validity evidence compared to other assessments. Integrating tools into training may help drive learners’ development and support competency decision-making.

**Please acknowledge all funding agencies by checking the applicable boxes below:**

CAG

**Disclosure of Interest:**

None Declared

